# Crystal alignment of caffeine deposited onto single crystal surfaces *via* hot-wall epitaxy†
†The authors declare no competing financial interest.
‡
‡Electronic supplementary information (ESI) available: Additional atomic force microscopy images and 2D FFT for various substrate temperatures; detailed X-ray pole figure analysis for needle growth on NaCl; optical microscopy images of caffeine grown on silica surfaces. See DOI: 10.1039/c7ce00515f



**DOI:** 10.1039/c7ce00515f

**Published:** 2017-04-27

**Authors:** Christian Röthel, Michal Radziown, Roland Resel, Andreas Grois, Clemens Simbrunner, Oliver Werzer

**Affiliations:** a Institute of Pharmaceutical Sciences , Department of Pharmaceutical Technology , Karl-Franzens Universität Graz , Universitätsplatz 1 , 8010 Graz , Austria . Email: christian.roethel@uni-graz.at ; Email: oliver.werzer@uni-graz.at; b Institute of Solid State Physics , Graz University of Technology , Petersgasse 16 , 8010 Graz , Austria; c Institute of Semiconductor and Solid State Physics , Johannes Kepler Universität Linz , Altenbergerstraße 69 , 4040 Linz , Austria; d Institute of Solid State Physics , University of Bremen , Otto-Hahn-Allee 1 , 28359 Bremen , Germany; e BioTechMed – Graz , Austria

## Abstract

Defined crystal growth is highly demanded for technological applications but also fundamental research. Within this work, the crystal growth of the asymmetric molecule caffeine was studied on single crystalline surfaces of muscovite mica, sodium chloride and potassium chloride. While elongated needle-like crystals grow on muscovite mica and sodium chloride, smaller individual “bird-like” structures were observed on potassium chloride. Depending on the surface type and temperature, the disk-shaped caffeine molecules prefer either an edge-on or flat-on orientation with respect to the surface, but in each case, a defined crystallographic relation between the surface and caffeine crystallites was determined by using the X-ray pole figure technique. On muscovite mica and sodium chloride, needle-like crystallites with edge-on oriented molecules aligned mainly with the unit cell *c*-axis (which coincides with the long needle axis) along the [1–10]_mica_, [100]_mica_, [110]_mica_ and [110]_NaCl_, [1–10]_NaCl_ directions, respectively. Crystals consisting of flat-on oriented molecules on KCl showed also defined alignments with respect to the substrate, but due to the altered molecule–substrate contact, the *b*-axis aligned along [110]_KCl_ and [1–10]_KCl_. Growth at elevated temperatures enabled changes in the crystal growth whereby more defined structures formed on NaCl. On KCl, the bird-like structures remained very similar, while caffeine on the mica surface at elevated temperatures resulted in even additional texture forming with the caffeine molecules now also favoring a flat-on orientation with respect to the surface. The systematic variation of various system parameters demonstrates how sensitive the growth behavior of caffeine on this variety of substrates is.

## Abstract 2

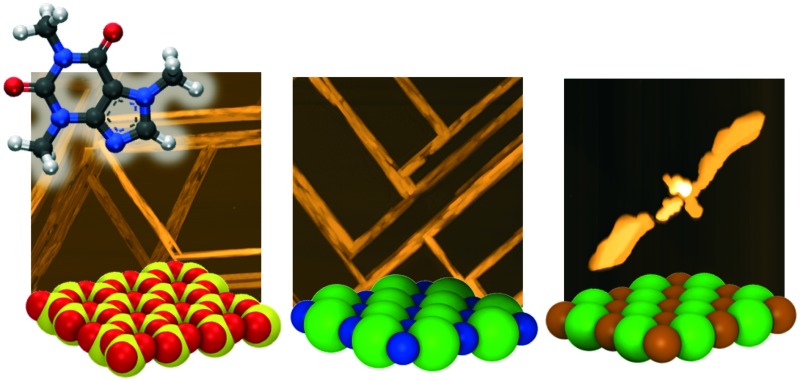
Crystal growth of caffeine on single crystalline surfaces yields needle or bird-like shaped crystals depending on surface chemistry and symmetry.

## Introduction

Controlled and specific crystal growth of organic materials is of high technological and fundamental interest as it promises reliable and, more importantly, reproducible properties demanded by research and applications.^1–3^ While often bulk solution processes are favored for their ease of use, for many technical applications crystallization directly on solid surfaces simplifies production. But the crystallization behavior in the vicinity of surfaces might be strongly influenced or altered compared to those in the bulk.^4,5^ As a simple example, the glass wall in a vessel already provides sufficient disturbance facilitating nucleation, and crystals grow more rapidly.^6,7^ In general, surface properties have a templating effect and often result in distinct crystalline forms.^8,9^ Isotropic surfaces like silica typically induce crystallization from defined contact planes, thus a uni-axial or fiber texture^10^ results, on which the azimuthal direction remains undefined.^11^ This results, for example, in caffeine needles growing along random directions on silica surfaces^12^ or pentacene forming randomly uncorrelated islands.^2^ Furthermore, surfaces are sometimes able to selectively dictate specific polymorph formation and additionally the alteration of the surface chemistry enables further tuning of the crystal growth, as observed for aspirin at polymeric surfaces.^13,14^ Besides the surface chemistry, the order and local morphology at the surface impacts crystal growth and nucleation.^15^ For instance, flat surfaces favor the formation of the bulk phase while rough substrates often lead to appearance of metastable thin film phases.^16^ The degree of order at the surface is larger if anisotropy is present, which also enables directional growth. A simple approach might be rubbed or drawn polymer films or more advanced techniques such as nanoimprinting in which surface corrugations facilitate the alignment of molecular crystals or liquid-crystalline polymers.^17–19^ Employing single crystal surfaces increases the degree of order even further and organic crystalline films might grow fully epitaxial.^20,21^ A few examples are sexiphenyl or caffeine grown on sheet silica^5,22,23^ or sexithiophene and perylene-derivatives on alkali halide surfaces.^24–26^ Epitaxial or directed crystal growth of organic crystals is used extensively in organic electronic devices or wave guiding applications,^27^ which both depend on a defined molecular orientation. In any case, grain boundaries and other crystal defects in such crystalline layers or needles are known to have a tremendous impact on the physical properties and performance.^28,29^ Moreover, epitaxial crystal growth has been used successfully in the selection of suitable crystal seeds and single crystalline surfaces to obtain elusive cocrystals or to control the polymorphism of active pharmaceutical ingredients.^30–32^ Epitaxial growth was also demonstrated to assist in the formation of theoretically predicted polymorphs such as carbamazepine form V.^33,34^

Within this work, we demonstrate the crystal formation of the model drug molecule caffeine on muscovite mica, sodium chloride (NaCl) and potassium chloride (KCl) surfaces. While the requirements on caffeine for pharmaceutical applications are mostly limited to purification and polymorph selection, this study offers interesting insights into the nature of organic crystal growth which are not accessible with frequently studied rod-like molecules.^26^ Firstly, in contrast to many rod-like molecules, caffeine is easily soluble in various organic solvents, which allows for a comparison between films grown by solution cast techniques and physical vapor depositions such as hot wall epitaxy. Secondly, caffeine molecules are disk-like but due to their chemical groups have a highly anisotropic character. This is stereotypical of many pharmaceutically relevant molecules, as especially their amphiphilic-like anisotropy allows for penetrating cellular membranes.^35^ Furthermore, caffeine exists in two polymorphic forms, whereby the stable β-form (CSD code: NIWFEE05) has an intriguingly large monoclinic unit cell which hosts 20 molecules. Moreover, molecules assemble in a slightly disturbed hexagonal motif, meaning the disk-like molecules have some degree of freedom in their orientation with respect to the neighboring molecules.^36–38^

Previous studies have shown that caffeine crystallites grow most often in the polymorphic β-form in the shape of extended needles but sometimes hexagonal structures on the account of the metastable α-form (NIWFEE04) are observed.^39^ It was recently shown that needles align on a mica surface when prepared by solution cast techniques.^12^ However, crystal growth during solution casting often takes place under non-equilibrium conditions with large variations in the growth kinetics. Thus, the crystalline alignment is rather undefined, which makes it difficult to draw conclusions. Conversely, using a hot wall epitaxy (HWE) process which operates closer to thermodynamic equilibrium^40,41^ was demonstrated to produce more defined and uniform crystal growth.^22^ In a typical HWE set-up, an independent adjustment of the source, wall and substrate temperature can be set ensuring optimal growth conditions for organic crystals. Moreover, as small molecules tend to evaporate rapidly under vacuum conditions, even at room temperature, depositions under ambient pressure conditions can be easily carried out by exchanging the ambient environment to an inert gas atmosphere.

In this work, we extend our previous investigations to various surfaces and temperatures, including NaCl and KCl providing different constraints for nucleation and crystal growth. Changes in the molecular adsorption geometry, crystal alignment and uniformity are evaluated using microscopy techniques. Differences in the directed crystal growth and epitaxial relations are identified by X-ray diffraction pole figure measurements and are correlated to specific surface properties and temperature effects.

## Experimental section

### Materials

Muscovite mica (grade V-4), NaCl as well as KCl substrates were purchased at SPI Supplies (West Chester, USA). Clean muscovite mica (001) surfaces were obtained by cleaving 15 × 15 mm mica sheets using adhesive tape. NaCl(001) and KCl(001) surfaces were obtained by cleaving the single crystals using a razor blade. After cleaving, the substrates were immediately transferred into the HWE chamber. A detailed description of the crystallographic properties of the substrate surfaces is provided elsewhere.^26^ Anhydrous caffeine of pharmaceutical grade was purchased from Herba Chemosan-AG (Vienna, Austria) and used without any modification.

### Sample preparation

A custom-made hot wall epitaxy chamber with independent ovens for the evaporation source (quartz tube), evaporation tube wall and substrate, was used to prepare various samples.^41^ The ambient atmosphere was exchanged with nitrogen immediately after sample transfer. Prior to deposition, substrates were equilibrated for 30 min at either 65 °C, 80 °C and 100 °C, ensuring stable thermal conditions. Subsequently, the evaporation source was heated and a shutter was opened so that the substrate surfaces were exposed to a molecular caffeine flux. The molecular depositions were performed under atmospheric pressure using a nitrogen atmosphere ensuring controlled and steady evaporation of the caffeine powder. The reliable operation temperatures for this study were 125° and 130 °C for the source and wall, respectively. An equal deposition time of 15 minutes was chosen for each type of substrate.

### Morphological investigation

Optical microscope images were obtained using an Axiovert 40 microscope (Zeiss, Germany) equipped with polarizers and a digital camera. The film topographies were investigated with a FlexAFM atomic force microscope equipped with an Easyscan 2 controller (both from Nanosurf, Switzerland). Height images were recorded in non-contact mode using a Tap190 cantilever (Budgetsensonors, Bulgaria) with a nominal resonant frequency of 190 kHz. All data were processed using the Gwyddion software package.^42^

### Crystallographic investigation

The crystallographic relations of caffeine needles and the substrates were investigated by X-ray diffraction pole figure measurements performed using a Philips X'pert diffractometer equipped with a chromium sealed tube, a secondary side graphite monochromator (*λ* = 0.229 nm) and optical slit systems. Briefly, an X-ray diffraction pole figure contains information about the spatial distribution of one specific net plane distance with respect to the substrate surface. During the measurement, the sample is continuously rotated around its surface normal (azimuthal angle *φ*) and tilted with respect to the incoming beam (inclination angle *ψ* of the surface normal) after a full 360° turn in *φ*. The diffraction intensity of each tuple (*φ*, *ψ*) is recorded and presented in polar contour plots using a stereographic projection, *ψ* being the polar radius and φ the polar angle. Spots of enhanced intensities (poles) correspond to Bragg reflections which are caused by net planes fulfilling the Laue condition, meaning the corresponding net planes are normal to the pole direction defined by *φ* and *ψ*. Simulating the recorded pole pattern using the software STEREOPOLE^43^ allows for the epitaxial relation to be deduced.

## Results

### Morphology

**Fig. 1:**
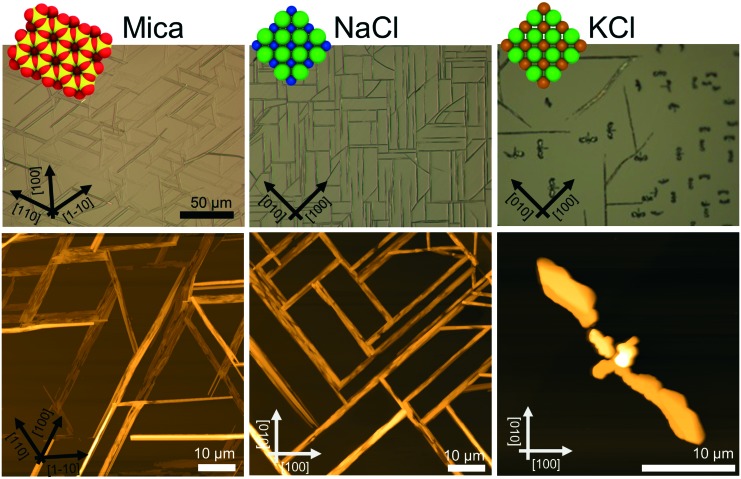
Top: Optical micrographs showing caffeine needles on various single crystalline surfaces. The inset illustrates the single crystalline surfaces made up of oxygen (red), silicon (yellow), chlorine (green), sodium (blue) and potassium (orange). Black arrows indicate the real space surface directions. Bottom: Atomic force microscopy height images showing the morphology of individual caffeine crystallites. Please bear in mind that AFM images are rotated with respect to the optical microscopy due to measurement constraints.

Caffeine molecules adsorb at a temperature of 65 °C onto all investigated single crystal surfaces (muscovite mica, NaCl, KCl) and form crystalline films with defined morphologies (see Fig. 1). On mica, needle structures develop, which run several 100 μm along the surface. The length only seems to be limited by other needles interfering so that the extension is limited due to geometrical constraints. A closer inspection of such a film using AFM height imaging reveals that these large needles are often a result of smaller needles packing closely together forming a fiber-like needle bundle. The typical width of such individual needles is approximately 200 nm and the height is 100 nm. The bundle-like structures have typical widths between 5 and 10 μm. Besides these, there are single needles which are thicker and higher compared to the others, suggesting that those are slightly different from those forming the broad bundles. However, for any of these structures there is a preferred alignment along the mica surface evident, *i.e.* they grow along three directions which are nearly parallel, 60° or 120° inclined to the lower image border. This is also reflected in the Fourier transform of the AFM image which reveals three streaks running along the same directions as observed in the height image (see the ESI‡).

Deposition of caffeine onto freshly cleaved NaCl surfaces results again in needle-like structure formation (see Fig. 1, middle). There is also a certain degree of bundle formation, but to a much lower extent. Compared to the mica sample, these structures have a smaller maximal width of 5 μm. The length again seems to be limited by adjacent needles interfering. The majority of needles show an alignment along distinct directions; needles aligning parallel or perpendicular to the lower image border exist, which is also reflected by FFT of the AFM images (see the ESI‡ Fig. S1). In addition, there are some needles inclining by about 45°. These needles seem to show a certain curvature, *i.e.* they deviate from a straight line. Such a curvature is absent in the other needles running 0° or 90° with respect to the image border. Furthermore, the inclined structures form only if the other needles are present, *i.e.* an independent alignment along this direction was not observed even if the caffeine deposition was significantly shorter (data not shown).

Exchanging the sodium in the surface by potassium, *i.e.* using a KCl substrate, the caffeine crystal morphology changes drastically. Occasionally, needle-like structures can be observed, which run along the same directions as on the NaCl surface, but the frequency of these structures is low. The more dominant morphologies are structures in a T-shape, even having similarities with the appearance of birds or bats with open wings, one might conclude. The wing span of the bird depicted in the AFM image (Fig. 1 right) is 26 μm and it has a head to tail distance of about 5 μm. The highest point is close to the wing ends and reaches nearly 3 μm. The individual birds align so that the tail points towards the same directions as the needles; this results in overall four different directions being noted, with birds looking up, down, left or right, respectively.

### X-ray diffraction pole figures

**Fig. 2:**
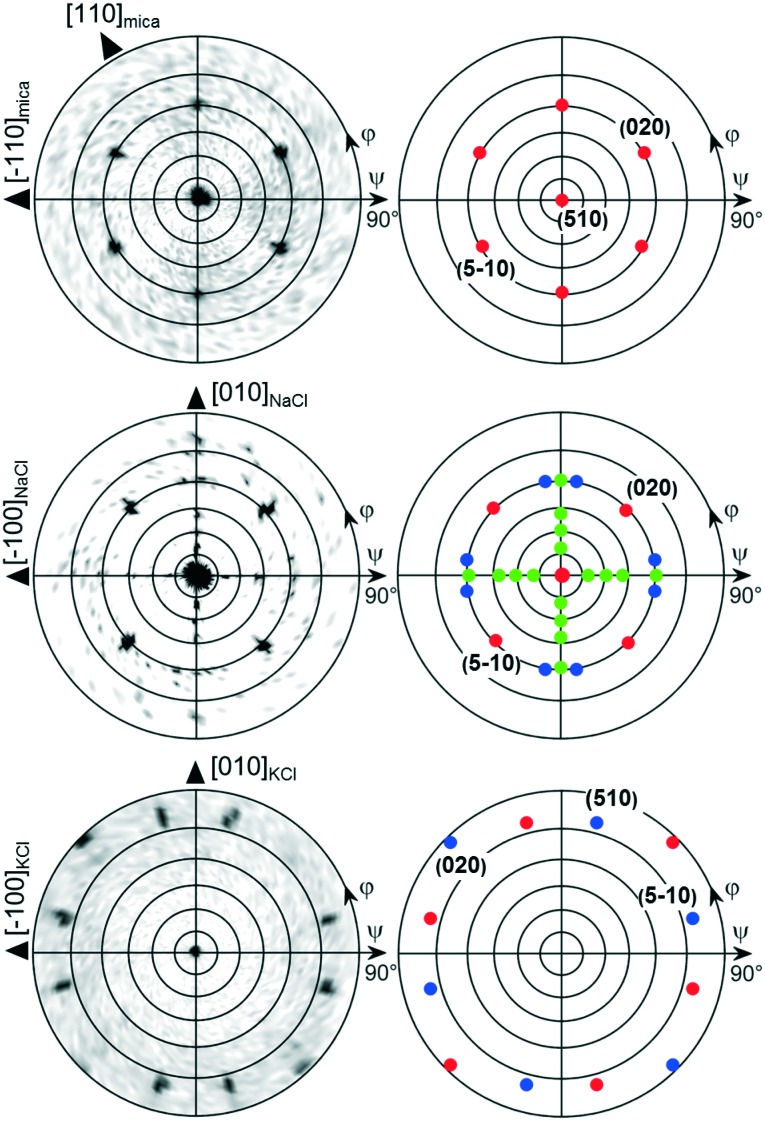
Pole figures used for the determination of the crystal alignment on muscovite mica, sodium chloride and potassium chloride single crystal surfaces. The left column shows experimental data and the right column shows the simulation. Black triangles indicate real space directions on the corresponding crystal surface.

The morphological investigations already suggest that directional defined caffeine crystal growth takes place at various surfaces. To extract the crystallographic information and to establish the epitaxial relations within the various samples, X-ray diffraction in the context of pole figure measurements is one of the best suited methods. Measurements for the various samples for a specific set of net planes are summarized in Fig. 2.

Starting with the mica sample, seven poles can be identified, all of which can be attributed to net planes with an interplanar *d*-spacing of approximately 0.75 nm (|**q**| = 8.4 nm^–1^). One pole is in the center which corresponds to net planes that lie parallel to the mica surface and is typically referred to as the contact plane. The remaining six poles are at inclination or tilt angles of *ψ* = 60° but vary in their azimuthal positions (*φ* = 30°, 90°, 150°, 210°, 270°, 330°). Setting the center of the pole figure to be the 510 reflection results in the poles at *φ* = 30° and *ψ* = 60° being the 020 reflection and the pole at *φ* = 210° and *ψ* = 60° corresponds to the 5–10 reflection. This means that one caffeine needle species is able to explain three poles. To explain the other poles at *φ* = 90°, 150°, 270° and 330°, two additional caffeine crystal alignments are required. These needles are identical in their crystallographic properties but their azimuthal alignment is rotated by 60° to another. Having knowledge of the crystallographic unit cell, the net plane orientations allow for the determination of the alignment of the crystal axes with respect to the surface. The orientation of the crystallographic axis of the substrate surface is established by measuring and indexing pole figures of easily accessible net planes of the substrate (data not shown) without changing the sample alignment in the diffractometer to obtain comparable measurements. For the first needle species at *φ* = 30°, the crystallographic *c*-axis points toward the [1–10] real space direction of mica.^22^ This direction coincides also with the long needle axis of one mica needle species. Hence, the *c*-axes (or long needle axis) of the remaining two needle species point towards the [110] or [100] mica direction.

This very same measurement was also performed on the NaCl sample (Fig. 2, middle). Again, the center pole can the attributed to net planes parallel to the surface, *i.e.* the (510). There are also poles found at inclination angles of *ψ* = 60° as on muscovite mica but the number of poles is reduced from six to four dominant peaks (red markers). These poles are found at *φ* = 45°, 135°, 225° and 315°. Since the measurement is taken at an identical scattering vector to that on the mica sample, a similar indexation scheme applies whereby only two needle directions require consideration to explain the dominant peaks. One crystal species is aligned with the *c*-axis pointing towards [110]_NaCl_ and the other points towards the [1–10]_NaCl_ direction. Besides the strong poles, some additional weak poles (green markers) are found along *φ* = 0°, 90°, 180° and 270° forming a cross-like shape which are accompanied by even weaker peaks (blue) to the left and right. The indexation reveals that these *c*-axes of these structures now point toward the [100]_NaCl_ and the [010]_NaCl_ directions, which is 45° rotated with respect to the previous. This direction is the same as in the intermediate needle structures observable off the two main directions.

As already expected from the microscopy investigations, the KCl sample shows a remarkably different behavior in the XRD measurements. The most striking difference in the pole figure (see Fig. 2, bottom) is the lack of a pole in the pole figure center (excluding the faint peak in the center due to tails of higher harmonic substrate peaks) and the region of *ψ* = 60°. In the case of KCl, twelve poles are located at inclination angles beyond 75°. They can be grouped into two sets of six poles (shown in red and blue), an indication of two crystalline alignments being present. Furthermore, such high inclination angles suggest that a change of contact plane took place. Since the net planes under investigation in this pole figure are still unchanged from the previous measurements, indexing the poles using 020 and 510 reflections identifies the alignment of the caffeine unit cell with the *b*-axis being along the [1–10] and [110] KCl surface directions.

### Substrate temperature variations

**Fig. 3:**
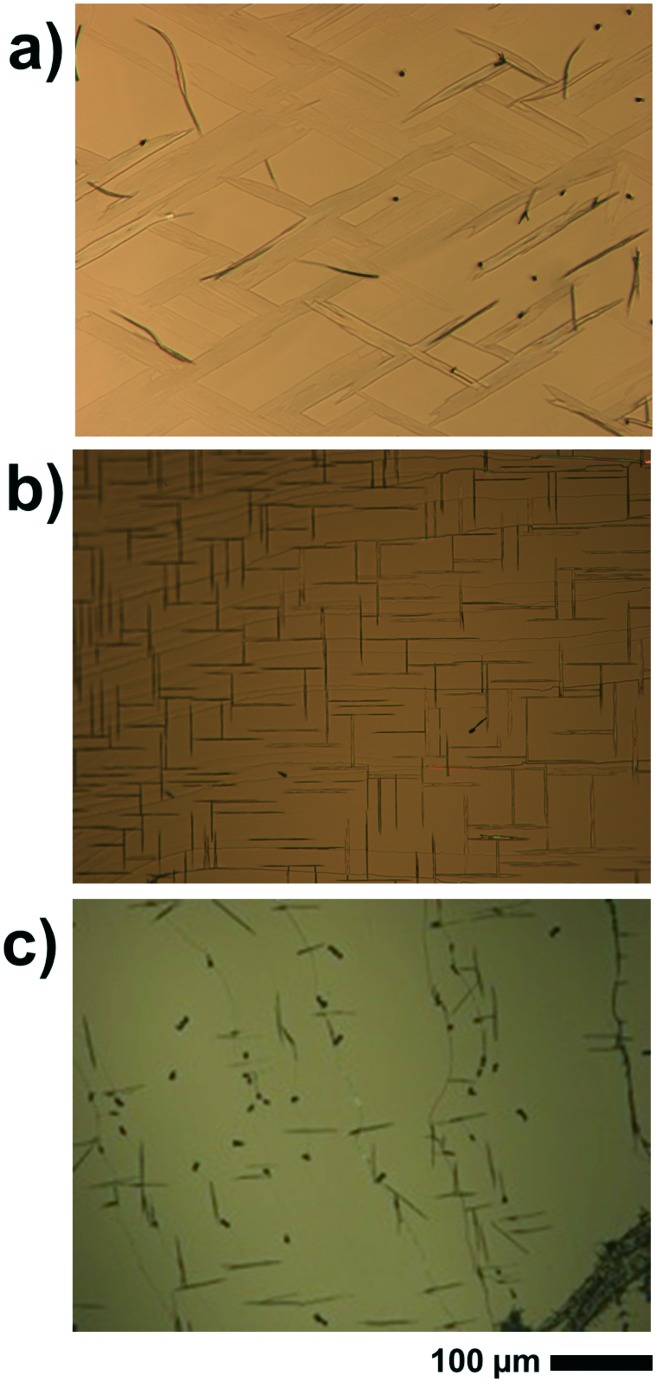
Optical micrographs showing the morphology of caffeine deposited at elevated substrate temperatures of 80 °C for muscovite mica (a) and 100 °C for NaCl (b) and KCl (c). The scale is identical for all images. Please bear in mind that the large object in the right bottom corner of (c) is caused by a cleavage step of the KCl crystal and not related to the caffeine deposition.

**Fig. 4:**
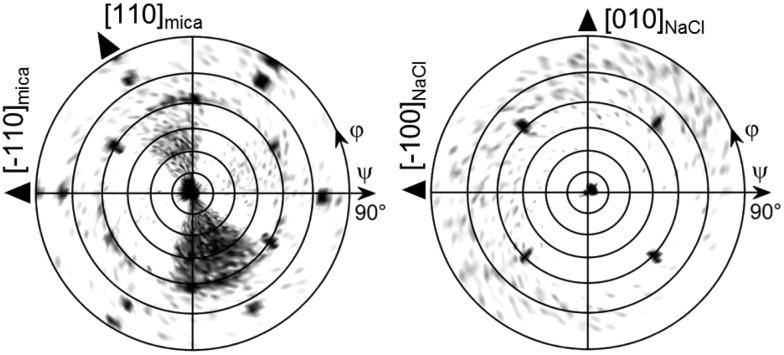
Pole figures of caffeine on mica (left) and NaCl (right) deposited at elevated substrate temperatures of 80 °C and 100 °C, respectively.

The deposition of caffeine onto mica held at 80 °C allows for adsorbing molecules and needle-like structures that form on the substrate surface (see Fig. 3a). Other than at lower temperatures, the order of the broader structures decreases significantly, resulting in needles showing some degree of curvature. A more detailed inspection shows that those broad structures consist of smaller needles of straight appearance packing slightly rotated to adjacent needles. Using a substrate temperature of 100 °C, caffeine molecules are unable to adsorb at the mica surface and hence a blank substrate surface resulted even after deposition as long as 20 minutes (data not shown). The pole figure measurement of the 80 °C sample contains poles identical to those of the 65 °C (see Fig. 4 left) *i.e.* six poles at inclinations of *ψ* = 60° are present. However, there are various other peaks which were absent at lower substrate temperatures. The indexation shows that these poles are explained by caffeine crystals of a different orientation (contact plane) which is very similar to the situation on KCl at 65 °C.

Moving on to the NaCl surface and using elevated temperatures shows again needle formation even for substrate temperatures as high as 100 °C, as shown in Fig. 3b. However, the sample differs from the NaCl samples prepared at 65 °C in two aspects. Firstly, the number of needles decreases but with slightly increased widths while leaving the height of the needles very similar at around 240 nm. The second difference is the absence of the intermediate structures which incline 45° to the others. Measurement at 80 °C shows an intermediate situation (see the ESI‡ Fig. S2), meaning the cancelation of the intermediate structures must take place in between 80 °C and 100 °C. The pole figure measurement, for a sample prepared at 100 °C, does show only information from the needles running along the [110] and [1–10] direction while the cross-like diffraction pattern vanished, as is somehow expected from the morphological differences (see Fig. 4, right).

A KCl surface allows for caffeine adsorption and crystal growth even at 100 °C too. Similar to the NaCl sample, the amount of caffeine on the surface decreases but the overall appearance of the structures remains nearly identical to the 65 °C sample (Fig. 3c). The pole figure investigation of a KCl sample prepared at 100 °C does not show any difference compared to those prepared at lower temperature (data not shown). Only the overall pole intensities are lower, which results from the lower amount of material present. Reliable conclusions regarding the relative amount of “bird”- and needle-like crystallites are difficult to draw since the intensity of the poles is strongly influenced by the sample roughness and macroscopic cleavage steps.

## Discussion

### Molecular orientation

**Fig. 5:**
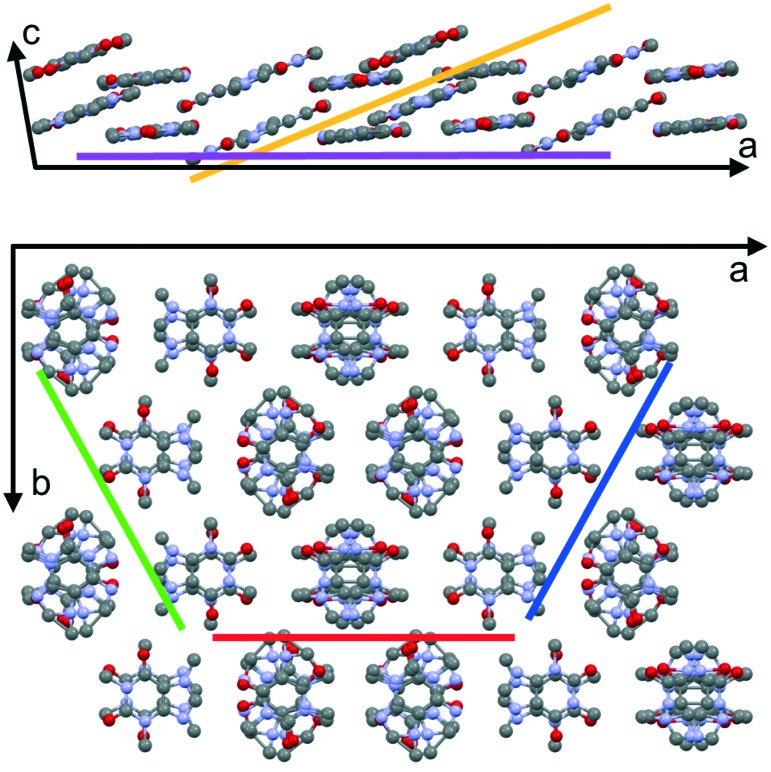
View along the crystallographic *b*-axis (top) and *c*-axis (bottom) of caffeine molecules assembling in the β-form. Projection of the (–703) and (001) net planes are shown in orange and magenta. (020), (510) and (5–10) net planes are shown in red, blue and green, respectively, and are arranged to highlight the hexagonal-like packing. Disorder is represented by overlapping molecules; hydrogen atoms are not shown.

Caffeine molecules are of disc-like shape and are highly asymmetric and feature a rather complex packing motif in the solid state. In the literature, there are two anhydrous polymorphs of caffeine described, the α and the β polymorphs, whereby in this study only the stable β-form was identified. In the β-form the molecules show parallel stacking along the crystallographic *c*-axis (see Fig. 5a). In the unit cell *a*–*b* plane, the molecules pack in a nearly hexagonal fashion, whereby a slight compression exists, as depicted in Fig. 5b. This compression means that molecules within the slices of the caffeine crystals incline slightly with respect to an adjacent slice resulting in an ABAB stacking order within the *a*–*b* plane. A particular interesting aspect of caffeine is its rotational molecular packing. Unlike in ideal or perfect crystal packings, some disorder disturbs the crystalline packing. While the centroid positions of the molecules are well-defined, the molecules exhibit rotational freedom around the disk plane normal (see Fig. 5).^38^ While the overall hexagonal-like packing motif remains unaffected, such disorder or plasticity means that molecules are more likely able to adapt to different adsorption sites rather than being constrained by the crystal lattice. This together with the large number of molecules within the unit cell means that likely molecular contacts with the substrate cannot be identified. From the experimental investigations however, the results of the collective caffeine interactions with the interface/substrate can be deduced, which for many applications are of high interest.

The particular characteristics of the caffeine crystal packing leads to the presence of various net planes of rather similar properties within the crystalline caffeine needle. Specifically, this means that crystal contacts with either the (020), (510) or the (5–10) plane are equivalently capable of describing the specific needle growth behavior at surfaces. Usually the crystal shape (morphology) results from low-energy planes (facets) which typically for crystals means that these differ for different contact planes. However, due to their similarity the morphology of individual caffeine needles is not affected by this very consideration of the actual contact plane. In fact, the occurrence of these three particular contact planes is just a result of needles being rotated by 60° along the long needle axis in accordance with the nearly hexagonal packing. This assumption is also reflected by the pole figure results which show a relative inclination of approximately 60° between the (020) and the (510) or (5–10) plane. Thus, poles with inclination angles of *ψ* = 60° will be observed regardless of which of the aforementioned contact planes is selected. Moreover, having one of these three contact planes as present on NaCl or some mica samples means that caffeine molecules adapt an edge-on molecular orientation with respect to the surface. This minimizes the interaction area with the underlying substrate.

For caffeine deposited either on KCl or on mica at 80 °C, poles in the XRD investigation appeared at inclination angles *ψ* beyond 75°. This is significantly higher compared to poles caused by edge-on oriented molecules which were at *ψ* = 60°. This inclination change has to be from crystal orientations in contact with net planes different from (020), (510) or (5–10), respectively; simulations of the data show that either a (001) or a (–703) contact plane results in these particular pole patterns. While the latter seems rather arbitrary, it becomes clearer on closer inspection of the unit cell and the molecular orientation therein (see Fig. 5). The (001) or (–703) contact planes provide cuts through the crystal with molecules lying parallel to these planes. Since the molecules within the unit cell can be separated into two groups (A or B stack) with different orientations, it seems reasonable that both contact planes provide similar adsorption properties due to their similarity regarding the actual molecule–substrate interaction. Comparing the pole inclinations resulting from these contact planes shows a theoretical difference of 5° in the inclination *ψ*, which should be sufficient to resolve two separate poles in the measurement. However, only one radially (*ψ*-direction) smeared out peak is observed. Hence, it is very likely that crystals adapt both the aforementioned contact planes, and even intermediate situations might occur, which explains one less defined pole. Despite some uncertainty in the contact plane determination, the overall absorption geometry changed clearly from an edge-on to a flat-on orientation with the change of substrate from NaCl to KCl or with increasing substrate temperature as in the case of muscovite mica.

Now the question arises as to why the caffeine crystals favor one orientation over the other. In general, there are several aspects to take into account. Molecules will adapt a flat-on orientation for the situation of molecule–molecule interaction being less dominant compared to the molecule–substrate interaction. In turn, strong molecule–molecule interaction in sufficiently large molecular clusters allows for a reorientation into an edge-on conformation as the amount of molecules at a surface increases.^26,44^ This mechanism is similar to the nucleation in solution as described by the two-step nucleation theory, where the actual nucleation is preceded by the formation of disordered clusters.^45,46^ Other reports demonstrated that the structure at the very interface region, which in our case is inaccessible, has a strong impact on the crystal formation/growth. For instance, the anisotropic surface properties favor molecular transport along certain directions,^47^ which then assists in nuclei formation consisting of lying molecules. On poor surface definition (or incompatibility), this nuclei formation required a molecule rising, so that molecule–molecule assembling into low energetic states is possible. Furthermore, one needs to keep in mind that caffeine has a plastic phase character too, meaning the molecules might choose their rotational order at some lattice sites. This, for instance, might allow that molecules rearrange to adapt for energetically more favorable adsorption geometries most likely required to adapt for the surface constraints.

### Azimuthal alignment

Besides the different molecular orientations with respect to the surface, caffeine also shows distinct azimuthal order on all substrates. For the edge-on caffeine crystals on mica, it follows that the *c*-axis, which coincides with the long needle axis, points towards the [1–10], [110] or [100] real space direction of mica when the substrate temperature was 65 °C during deposition. This particular alignment is clearly connected to the surface geometry of muscovite mica. Mica is a rather complex material and belongs to the sheet silicate family featuring a layered structure of two alternating layers.^48^ Cleaving separates two (001) planes, leaving this plane as an outer surface for molecule deposition. Such a surface features a regular lattice which is defined by oxygen atoms running along three distinct directions inclining 120° to one another. This would suggest that mica has a 3-fold symmetry. However, intermediate places in the surface are occupied by aluminum atoms. These are ordered differently compared to the oxygen atoms, which causes a slight surface lattice distortion with oxygen atoms being slightly lower along the [1–10] direction. Thus, a corrugation exists in the surface with an approximate depth of 0.08 nm, as determined by AFM investigations.^49^ As a consequence, the mica surface features only mirror symmetry along the [1–10] direction.^5^ Depending on the deposit, mica enables order due to its mirror symmetry or *via* its pseudo 3-fold symmetry.^5,22^ In fact, the caffeine needles in this study align along three directions in accordance with a pseudo 3-fold symmetry. Also the long needle axis coincides with the hexagonal-like packing of the oxygen atoms in the mica surface. As pointed out earlier, the molecules in such a caffeine needle show various orientations with respect to the surface. This suggests that the alignment during the nucleation or growth process likely results from collective adaptation of many molecules simultaneously. This is distinct from systems of strong single molecule–substrate interactions for which the crystal alignment is dominated on a smaller scale, *i.e.* the molecule rather than the collective nuclei/crystal.^5^

It should be noted, that deposition of caffeine on mica, even just for very short times of 5 minutes (data not shown) always shows three directions being present, *i.e.* needles growing along a certain direction are always accompanied by other needles which are inclined by 60°. Interestingly, 60° inclination can be observed on NaCl surfaces as well even if it is only obvious when close to other needles. For samples prepared on isotropic silica surfaces, this 60° branching also exists (see the ESI‡ Fig. S3) which means that this type of structure is likely an inherent property of caffeine crystals growing at surfaces and is often referred to as ledge directed growth.^50^ Ledge directed growth means that a facet on a crystal is able to host nuclei of different crystal directions, which eventually grows into needles, in the case of caffeine, 60° off the initial direction. While this effect is much less frequent on NaCl, the pseudo 3-fold symmetry which exactly matches the 60° inclination might assist in the ledge directed growth, which in turn means that the other directions are more likely to form. If the other directions are a consequence of solely ledge directed growth, the initial alignment of the caffeine “network” might likely occur due to the surface corrugation along the mirror plane direction of mica, *i.e.* the [1–10] direction, and the others are just coincidently aligned along the other main mica directions.

**Fig. 6:**
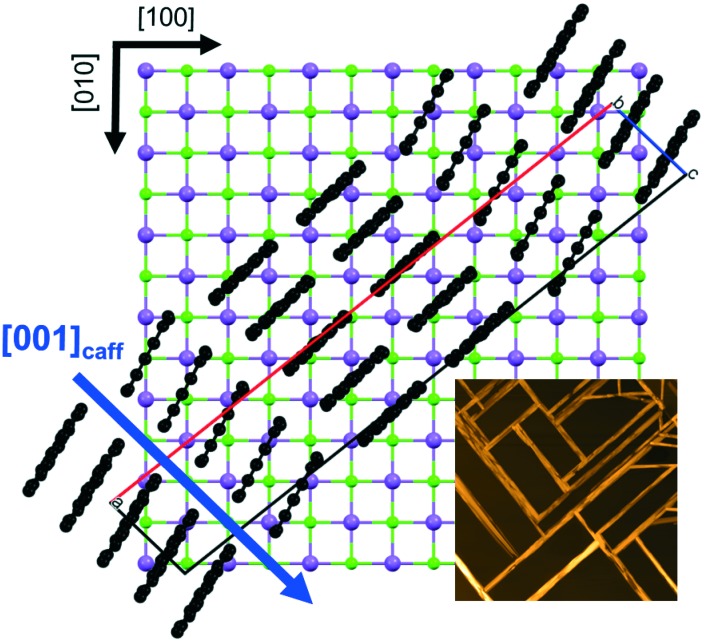
Molecular alignment of caffeine with respect to the NaCl(001) surface depicted for caffeine needles growing along the [110]_NaCl_ direction. The blue arrow indicates the long needle axis of caffeine.

On NaCl, caffeine needles align with the *c*-axis along the [110] and [1–10] direction, while the molecules adopted an edge-on orientation which coincides with the surface corrugations on the NaCl surface (c.f. Fig. 1 and 6). The grooves in the surface result from the different ion radii of sodium (0.116 nm) and chlorine (0.167 nm), giving an approximate corrugation depth of 0.05 nm which is slightly smaller than the grooves on muscovite mica (0.08 nm). Besides the obviously altered substrate surface chemistry, this geometrical constraint (corrugation) may also play an important role in the overall needle alignment. A similar behavior was found previously in other systems, where surface corrugations in alkali halide surfaces induced directed growth of sexiphenyl or perylene-derivatives such as PTCDA among others.^25,51^ Nevertheless, *c*-axis alignment along the surface grooves means that the caffeine disks preferably align along rows consisting of either sodium or chlorine rather than with alternating atom species, as schemed in Fig. 6.

The morphological investigations reveal that for the NaCl sample needles along [110] and [1–10], and also needles which align partly along the [100] or [010] direction are present. As briefly mentioned before, the growth of such needles is very likely a result from ledge directed growth, *i.e.* needles start out at a facet of a “regular” substrate aligned needle and grow with inclinations of 60°, as can be seen in the AFM investigations. On further extension from the facet, the needle direction bends and eventually results in a needle alignment of 45° with respect to the other crystal directions. This behavior might reflect the competing impact of the crystal and the substrate on the needle alignment; the regular needle dictates the initial growth process while the underlying substrate tries bending the needle for more favorable positions and a gradual transition (curvature) results. In addition to the visible morphology, the cross-like feature in the pole figure with various poles along a given *φ* angle means (green markers for *ψ* < 60°) that these inclined structures have some degree of freedom in their contact plane, again in agreement with the hexagonal nature of the caffeine packing in the unit cell. This results in many rotations around the long needle axis (*c*-axis) forming simultaneously on the sample surface.

Further analysis of this complex pole figure regarding the accompanying needles (blue poles) revealed that an *a*-axis alignment along the [100] or [010] also exists. Such an *a*-axis alignment can be easily seen because of the monoclinic caffeine unit cell in which the growth directions deviate by ±9° with respect to the *c*-axis aligned needles (a detailed explanation is provided in the ESI). This means that on NaCl there is also a favorable energetic state (local energetic minimum) which then results in a slight deviation of the interaction with the substrate. Nevertheless, 9° inclination might be just a result of adjacent caffeine sheets being inclined by a certain degree, meaning that a caffeine A-layer is about 9° rotated to a B-layer. A substrate temperature of 100 °C during deposition suppresses the growth of any ledge directed needles. Elevated substrate temperatures mean that more energy is present in the system, which allows for escaping the local minima required for ledge directed growth more easily. Thus, molecule arrangements in energetically more favorable positions are solely occurring.

Despite being in the same crystal class (rock salt) and thus providing a similar cubic surface geometry the resultant crystal growth on KCl and NaCl is remarkably different. Due to the substitution of sodium with potassium, the cubic lattice constant increases from *a*_NaCl_ = 5.64 Å to *a*_KCl_ = 6.29 Å. Since the ion radius of potassium (0.152 nm) is considerably larger than of sodium (0.116 nm), the surface corrugations along the KCl surface decreased significantly compared to the NaCl surface grooves. Moreover, due to the increased KCl lattice spacing the lateral distance between adjacent surface grooves increases. This is of particular interest if compared to the *c*-axis of the caffeine crystal, which is 6.95 Å (see Fig. 6). Similar to lattice matching, as used for inorganic epitaxy, the compatibility of caffeine with the substrate can be estimated; the lateral groove spacing on KCl is *d*_g,KCl_ = 4.45 Å and on NaCl this spacing decreases to *d*_g,NaCl_ = 3.97 Å. Using a simple calculation, it was shown that 7 × *d*_g,NaCl_ Å = 4 × *c*_caffeine_ ± 0.05 Å, whereby a similar calculation for caffeine on KCl did not provide any similar agreement (the closest match is obtained for 11 × *d*_g,KCl_ Å = 7 × *c*_caffeine_ ± 0.23 Å which has a much larger error). From this simple consideration, it can be followed that the NaCl lattice along the considered direction is more compatible with edge-on molecular caffeine packing if compared to the KCl lattice for which the flat-on molecules are favorable.

The consequence of the flat-on molecule orientation on KCl reveals morphologies which deviate strongly from elongated needle shapes. Crystals of a bird-like character, containing wings and a tail, are observed. More detailed inspection might even allow suggesting that the wings of the birds are composed of facets inclined by 120°, corresponding to hexagon formation together with some linear (needle-like) segments forming the bird tails. Pole figure measurements show clearly a defined crystal alignment with respect to the KCl substrate surface where the *b*-axis is in-plane with the surface, aligning either along the [110]_KCl_ or the [1–10]_KCl_ direction. The molecular packing in the unit cell *a*–*b* plane is hexagonal, which is similarly oriented to the 120° segments of the bird wings. Remarkably, the individual birds have a similar size on the entire substrate surface meaning a well-defined growth mechanism is responsible for this bird formation. For instance, a single bird wing is not symmetric, and the upper side seems different from the lower side. Likely, this is a result of the crystal *c*-axis being not perfectly perpendicular to the surface, thus also the *b*-axis is slightly inclined with respect to the sample surface. On molecule condensation at the crystal sites, this inclination favors crystal growth along one direction while simultaneously disfavoring the growth in the other direction simply due to geometrical constraints.

## Conclusion

While defined needle-like crystal growth on solid substrates is often observed for organic molecules, disk-like caffeine with its asymmetric molecular conformation shows a particularly complex growth behavior. While elongated needle-like crystallites consisting of edge-on oriented molecules run along characteristic surface directions on sodium chloride and muscovite mica, smaller “bird-like” structures are observed on potassium chloride due to flat-on oriented molecules. However, on every single crystal surface investigated in this study an epitaxial relation between the surface and the caffeine structures could be identified. The differences in the various samples in terms of caffeine growth mean that the molecular orientation as well as the azimuthal alignment depends strongly on the substrate surface geometry and chemistry but also on the substrate temperature during deposition. Interestingly, caffeine crystallites align well at the surface whereby the crystals have significantly more freedom compared to other organic crystals, *i.e.* many different contact planes allow for a very similar macroscopic needle alignment. Many aspects of its intriguing growth behavior are most likely rooted in its crystalline packing which offers some orientational degrees of freedom for the molecules within the crystal unit cell but also for the adsorption sites. This means that the crystal growth and alignment is likely driven by the collective interaction properties of a caffeine crystal with the surface, rather than a result of single molecule–substrate interactions. The results of this study clearly show that the molecular orientation with respect to the surface might change (different contact planes) but the crystalline alignment can be conserved. To some extent it might be possible to find a similar behavior for a variety of disc-like molecules, for which specific growth is of high interest whereby it is very likely that the chemistry of the molecules, the preparation technique and all substrate properties will have a profound effect on the directional growth.
