# Supplementary material for: Crystal alignment of caffeine deposited onto single crystal surfaces via hot-wall epitaxy
Source: CrystEngComm. 2017 Apr 27;19(21):2936–45. doi: 10.1039/c7ce00515f (PMC5471919; doi:10.1039/c7ce00515f)
Supplement: Supplementary file 1 [file CE-019-C7CE00515F-s001.pdf]

Electronic Supplementary Material (ESI) for CrystEngComm.
This journal is © The Royal Society of Chemistry 2017

Electronic Supplementary Information

Crystal alignment of caffeine deposited onto single crystal surfaces via hot-wall-epitaxy

Christian Röthel,†§ Michal Radziown,‡ Roland Resel, §¥ Andreas Grois,‡ Clemens Simbrunner,‡¶ and Oliver Werzer*†¥*

[†]Institute of Pharmaceutical Sciences, Department of Pharmaceutical Technology, Karl-Franzens Universität Graz, Universitätsplatz 1, 8010 Graz, Austria

[§]Institute of Solid State Physics, Graz University of Technology, Petersgasse 16, 8010 Graz, Austria

[‡]Institute of Semiconductor and Solid State Physics, Johannes Kepler Universität Linz, Altenbergerstraße 69, 4040 Linz, Austria

[¶]Institute of Solid State Physics, University of Bremen, Otto-Hahn-Allee 1,

28359 Bremen, Germany

[¥]BioTechMed – Graz, Austria

Atomic Force Microscopy

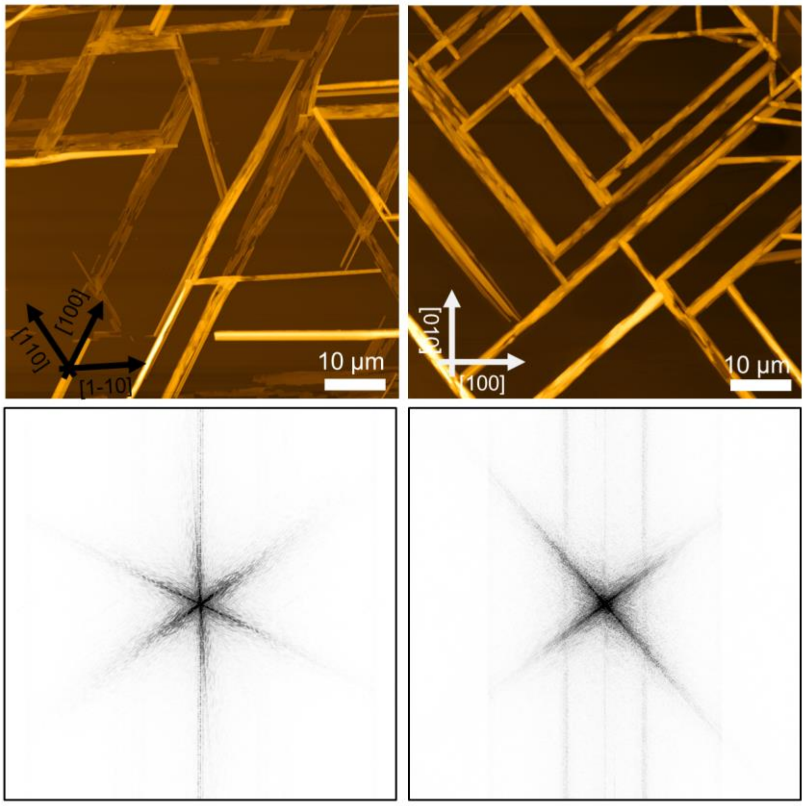

Sup. Figure S1. Atomic force microscopy height images of caffeine needles on muscovite mica (left) and sodium chloride (right). Bottom row shows the corresponding 2D FFT reflecting the 3-fold and 2-fold symmetry of the needle growth on muscovite mica and NaCl surfaces, respectively.

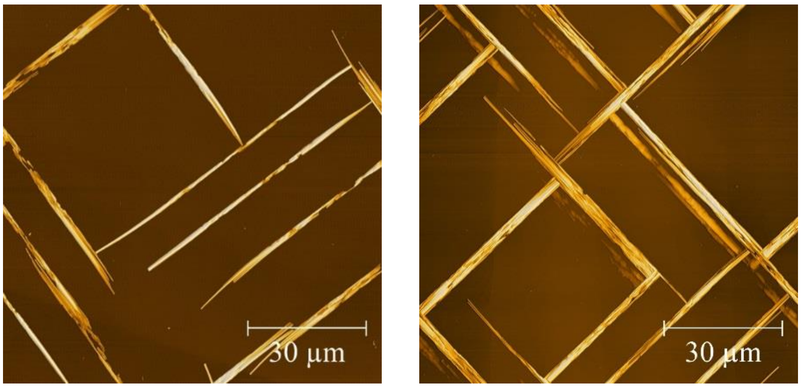

Sup. Figure S2. Comparison of AFM height images showing caffeine needles deposited on NaCl at 100°C (left) and 80°C (right). Intermediate directions along [100] and [010] i.e. rotated by 45° with respect to the depicted needles, are not observed due to the elevated substrate temperatures.

<u>Caffeine on silica surfaces</u>

Caffeine grown on isotropic silica surfaces results in short needle-like structures, with the size and coverage depending on the substrate temperature (see Figure S3). In contrast to caffeine grown on single crystalline surfaces, the needles show no particular azimuthal alignment. While the needles itself are randomly distributed along the surface, contacting needles show a preference for inclination angles of approximately 60° or 120°, respectively.

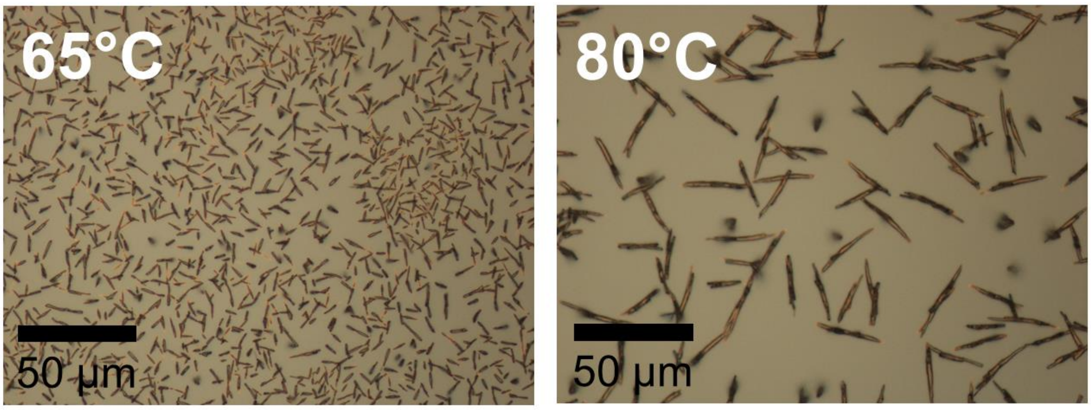

Sup. Figure S3. Caffeine grown on isotropic silica at 65°C and 80°C substrate temperature.

[100] and [010] needle alignment on NaCl

While samples prepared at 100°C on NaCl show a well-defined crystal growth along the [110] and [-110] direction, the growth at lower temperatures of 65°C shows a more complex and diverse crystal growth. The corresponding X-ray pole figure and the result is shown in Figure S4. Most pronounced are the specular reflection at ψ = 0° and four peaks at ψ = 60° with φ = 45°, 135°, 225° and 315°. These are the very same positions as obtained at 100°C substrate temperature (see Figure 4 in the main text), i.e. these correspond to needles grown along the $[110]_{NaCl}$ and $[1\text{-}10]_{NaCl}$ direction, respectively. In the indexation pattern these are indicated by red dots (see Figure S4). Other poles at ψ = 60° are found but with φ = 0°, 90°, 180° and 270° (green dots). Their intensities are low, meaning that these are present less frequently. For these azimuthal alignments (φ) also poles at different inclinations ψ are found; low intense poles are located at ψ = 20°, 31° and 42°. While this seems to be a very complex pole figure, the caffeine packing allows understanding this pole figure in detail. Starting with the "green" pole at (ψ/φ) = (60°/90°): It is explained by caffeine having again a (510) contact plane. Other than on the sample prepared at 100°C, the needle axis is rotated around φ by 45°, which means the long needles axis points towards the $[010]_{NaCl}$ direction. Poles at ψ = 20°, 31° and 42° are a consequence of caffeine needles with altered contact planes: (520), (530) and (550) contact planes explain these poles. These contact planes share a common axis of rotation (c-axis) known as the zone axis i.e. the needles are rotated along the long needle axis while their azimuthal alignment φ remains unaffected. Nevertheless, there are still some unattended weak residual poles (blue dots) left. Firstly, assuming a (020) contact plane results in the (510) and (-510) being the corresponding poles to index these reflections. The indexing of these poles results in the c-axis inclining by 9° with respect to the $[100]_{NaCl}$ direction but now with the a-axis matching $[010]_{NaCl}$ (or equivalent directions). While this might be surprising, the reason for such a splitting on account of the a-axis alignment is due to the

monoclinic unit cell angle β = 99°. Thus, flipping a crystal with a (020) contact plane creates a (0-20) contact plane and the apparent monoclinic angle changes to 81°, meaning the crystal flipping is not invariant.

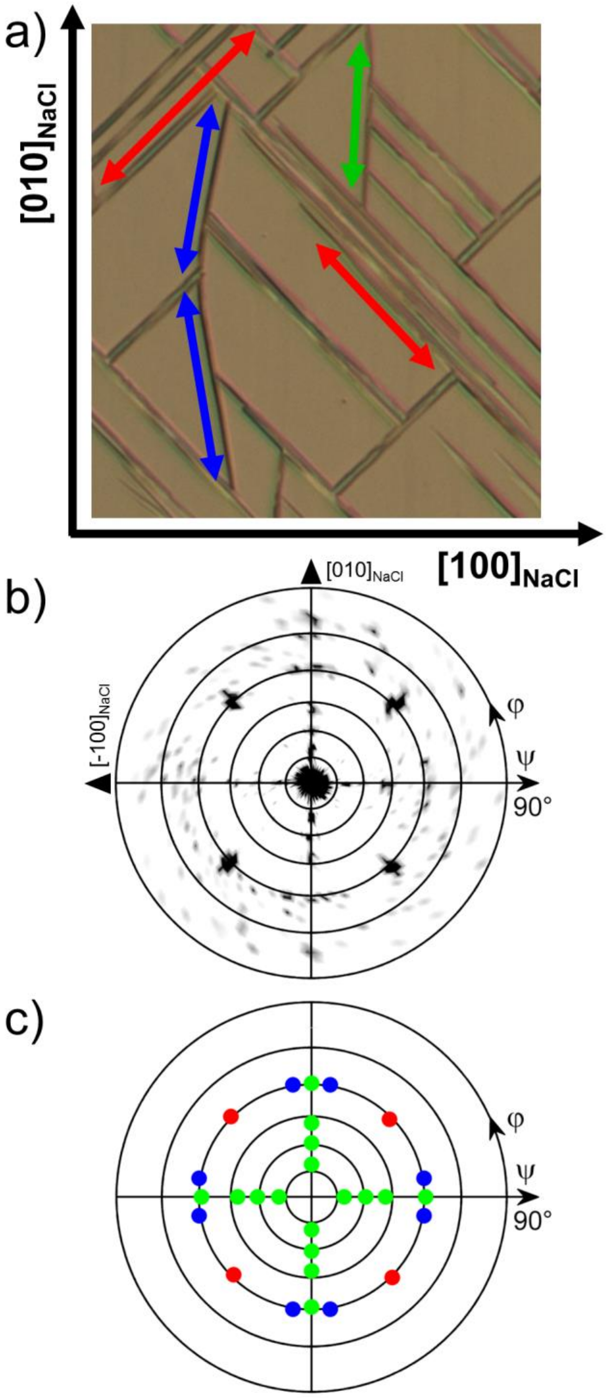

Sup. Figure S4. a) Optical micrograph of needles grown on NaCl at 65°C substrate temperature. Black arrows indicate NaCl real space directions and colored arrows indicate the

long needle axis (caffeine c-axis) for various needle alignments. Corresponding pole figure measurement taken at q = 8.4 nm^{-1} (b) with simulation (c). Black triangles in the pole figure indicate NaCl surface directions.
